# Nano to micro delivery systems: targeting angiogenesis in brain tumors

**DOI:** 10.1186/2040-2384-2-20

**Published:** 2010-10-08

**Authors:** Ariel Gilert, Marcelle Machluf

**Affiliations:** 1 Faculty of Biotechnology and Food Engineering, Technion Israel Institute of Technology, Haifa, Israel

## Abstract

Treating brain tumors using inhibitors of angiogenesis is extensively researched and tested in clinical trials. Although anti-angiogenic treatment holds a great potential for treating primary and secondary brain tumors, no clinical treatment is currently approved for brain tumor patients. One of the main hurdles in treating brain tumors is the blood brain barrier - a protective barrier of the brain, which prevents drugs from entering the brain parenchyma. As most therapeutics are excluded from the brain there is an urgent need to develop delivery platforms which will bypass such hurdles and enable the delivery of anti-angiogenic drugs into the tumor bed. Such delivery systems should be able to control release the drug or a combination of drugs at a therapeutic level for the desired time. In this mini-review we will discuss the latest improvements in nano and micro drug delivery platforms that were designed to deliver inhibitors of angiogenesis to the brain.

## Introduction

It is now evident that solid tumors beyond a given volume are dependent on the supply of oxygen and nutrients from the vascular system, which has to grow concomitantly with the tumor, similar to embryonic development. This process, of newly developed blood capillaries and blood vessels from pre-existing ones, has been termed angiogenesis and enables the tumor not only to increase its size but also its aggressiveness and its ability to metastasize [1-4]. The process of angiogenesis is implicated not only in the pathology of tumors but also in many other diseases including psoriasis [5,6], age-related macular degeneration[7,8] and rheumatoid arthritis [9].

**Figure 1:**
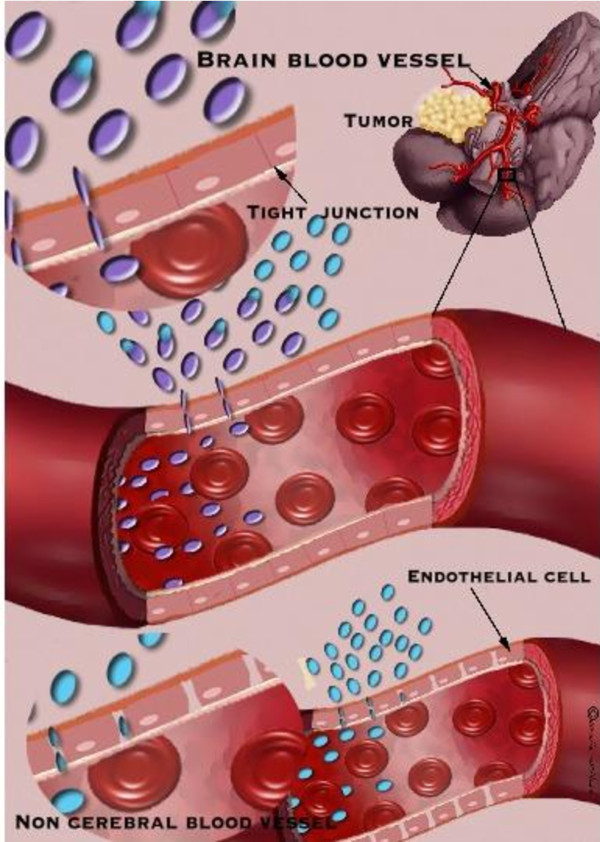
**Comparison between cerebral and noncerebral blood vessels**. Cerebral blood vessel has tight junctions, which do not allow the passage of un-desirable and pathological substances to the brain parenchyma while non cerebral blood vessel allows better diffusion of drugs. Targeting the brain can be achieved using the drug itself or by using drug delivery platforms that release the drug at a specified location.

Some of the most deadly malignancies that depend on the angiogenic process for their growth are primary brain tumors[10], among which glioblastoma multiforme (GBM) represents 40% of all cases. GBM has been targeted with many inhibitors of angiogenesis including tissue inhibitors of matrix metalloproteinases[11-13], chemokines [14-16], tyrosine kinase inhibitors [17-20], interleukins [21,22], and naturally occurring proteolytic fragments of large precursor molecules such as endostatin, vasostatin, canstatin, angiostatin and others [23-29]. These molecules exert their inhibitory functions on endothelial cells by multiple mechanisms including proliferation, migration, protease activity, as well as the induction of apoptosis [30]. Although such angiogenesis inhibitors hold great promise, the ones that reached clinical trials for brain tumor patients have failed to achieve significant therapeutic outcome. One possible explanation for this outcome that is supported by many researchers is the lack of combinatory treatment with standard chemo and radiotherapy [31]. Another obstacle which may hamper the therapeutic outcome of anti-angiogenic therapy is the blood brain barrier (BBB, although destabilized in high grade GBM patients) which therapeutics need to bypass to exert a significant brain tumor inhibitory effect. The brain vasculature is predominantly different that of other tissues as its principal role is to prevent un-desirable and pathological substances from entering the brain parenchyma (Figure 1) [32]. The physical properties of the BBB, which include continuous tight junctions and low pinocytotic activity as well as high electrical resistance (attributed to occludin expression), form a tight barrier against materials with high molecular weight and ionic substances that can enter the brain parenchyma only through active transport [33,34]. As such, the BBB hampers and complicates the systemic delivery of therapeutics to the brain [35,36]. Small lipophilic drugs, which are expected to diffuse across the BBB, are removed from the central nerve system (CNS) by efflux transporters, such as P-glycoprotein (P-gp) [32,37]. Other drug-based transporters that enable multi-drug resistance include the multi-drug resistance-associated protein (MRP) family (MRP1-MRP9) expressed in brain endothelial cells, breast cancer-resistant protein (ABCG2) [38,39] and organic anion and cation transporters (OAT and OCT respectively) [40,41].

Nonetheless, it is also possible that the method of administration, which may destabilize the drugs, the therapeutic level needed to reach the tumor or a combination of all the above hurdles contribute to the disappointing outcome of such therapy approach.

These obstacles have resulted in a more urgent focus on developing alternative delivery modalities, which may bypass the BBB and efficiently target tumorangiogenesis while protecting and stabilizing the drug until reaching the tumor bed. These delivery modalities, may not only solve the problem of the BBB permeability and the need for a combinatorial treatment, but also reduce the therapeutic amount of drug needed to be delivered to the tumor, thus lowering toxicity and side effects of the drugs. Nevertheless such delivery modalities, whether local or systemic, have to deliver the therapeutics to the tumor mass, where the diffusion and distribution of the drug is governed by abnormal high tumor cell density, high interstitial fluid pressure within the tumors and leakiness of tumor microvasculature, resulting in fast clearance of the diffusing drug [42,43].

In this review, we will discuss the progress made in designing and developing different nano and micro drug delivery platforms that aim to bypass the BBB and deliver systematically or locally therapeutics that target brain tumor angiogenesis. Table 1 summarizes the current clinical status of therapeutics, which are also utilized in studies aimed to develop drug delivery platforms for brain tumor therapy as will be discussed later on.

**Table 1:** FDA approved or under clinical trails drugs for brain tumors therapy

Drug	FDA approved for brain tumors/Brain clinical trails	Approved routes of administration (Brain tumors & other disorders)	Drug delivery platforms in research for approved drugs
Temozolomide	Approved	Oral	Intracerebral Biodegradable gel matrices/Polymer nanoparticles
Procarbazine	Approved	Oral	-
lomustine	Approved	Oral	Liposomes/Microcapsules
Vincristine	Approved	IV	Intra-arterially
Carmustine	Approved	IV/Oral/Wafer	CED/Polymer microchips and microspheres
Carboplatin	Approved	IV	CED/Intracerebral/Intraarterial/Liposomes
Bevacizumab	Approved	IV	Intra-arterial
Doxorubicin	Phase I/II/III	injection;liposomal *	-
Imatinib mesylate	Phase I/II/III	Oral; Intravenous *	-
Cisplatin	Phase I/II/III	Injection *	-
Topotecan	Phase I/II/III	Injection; Oral *	-
Interferon-alpha	Phase I/II	Injection; Subcutaneous; Oral *	-
Paclitaxel	Phase I/II	Intravenous; Injection *	-
Arsenic trioxide	Phase I/II	Injection *	-

* These routes are approved for other disorders

## Local drug delivery platforms

Local delivery to the brain utilizes BBB disruption, as well as local implantation of the delivery system directly in the tumor bed. These delivery systems which include cerebral infusion methods, polymeric nano-particles, wafers and more, are comprehensively studied and represent promising approaches for the delivery of drugs to the brain.

### Intra-arterial cerebral infusion

Intra-arterial cerebral infusion involves the insertion of micro-catheters into the small arteries of the brain via the carotid artery [44]. This unique approach was used to infuse mannitol into the area of interest for transient disruption of the BBB, followed by the infusion of a therapeutic such as bevacizumab. As bevacizumab is selectively delivered to brain tumors, larger amount of the drug may be used when compared to the amount of drug used by intravenous administration of bevacizumab resulting with reduced side effects [44]. This delivery system may also enable the direct delivery of other drugs, which target the angiogenic processes in brain tumors.

### Polymeric particles

Another popular approach, which is still in preclinical studies, is based on polymeric nano and micro-particles using polymers such as poly(butyl cyanoacrylate), Poly(ethylene-glycol), Poly(lactic-co-glycolic) acid, Poly-glycerol and others [45-48]. These particles can be loaded with or attached to different therapeutics and can then be delivered directly to the tumor site. The use of particle platforms significantly reduces (in some cases more than 50 folds) the amounts of drugs needed to reduce tumor volume and weight when compared to systemic administration. Such platforms need to be carefully designed, taking into account the properties of the drug carrier in term of immunogenicity, stability, preparation method, manufacturing costs, biodegradability and its pharmaceutical qualities (stability of the therapeutic, dosage capability, distribution and site specific targeting). These delivery vehicles must also retain the biological activity of the drug and allow its sustained release over extended periods of time when needed [49]. This is particularly important when attempting to deliver endogenous inhibitors of angiogenesis as opposed to chemotherapeutic drugs.

### PLGA particles

One of the widely used polymers for the design of different delivery platforms is the poly-lactic-co-glycolic based polymer (PLGA). The huge advantages of delivery formulations based on PLGA, are their non immunogenicity and the ability to control the release profile of the drugs by manipulating the ratio of lactic to glycolic acids. An interesting publication by Shahani *et al*. showed that curcumin encapsulated in PLGA microspheres down regulates markers of angiogenesis such as CD31 and vascular endothelial growth factor (VEGF) in nude mice bearing MDA-MB-231 xenografts. Furthermore, curcumin levels in the brain were 10 to 30 folds higher than in blood indicating the possibility to use this formulation to treat brain tumors [50]. Arai *et al*. showed the feasibility of using thermoreversible gelation polymer combined with doxorubicin loaded PLGA microspheres or liposomes for local treatment of malignant glioma [51]. PLGA microspheres have also been used to carry glioma cell lysates for the induction of protective immunity in rat glioma model. Although this system was less efficient than irradiated cell lysate it does exhibit adjuvant properties [52].

**Figure 2:**
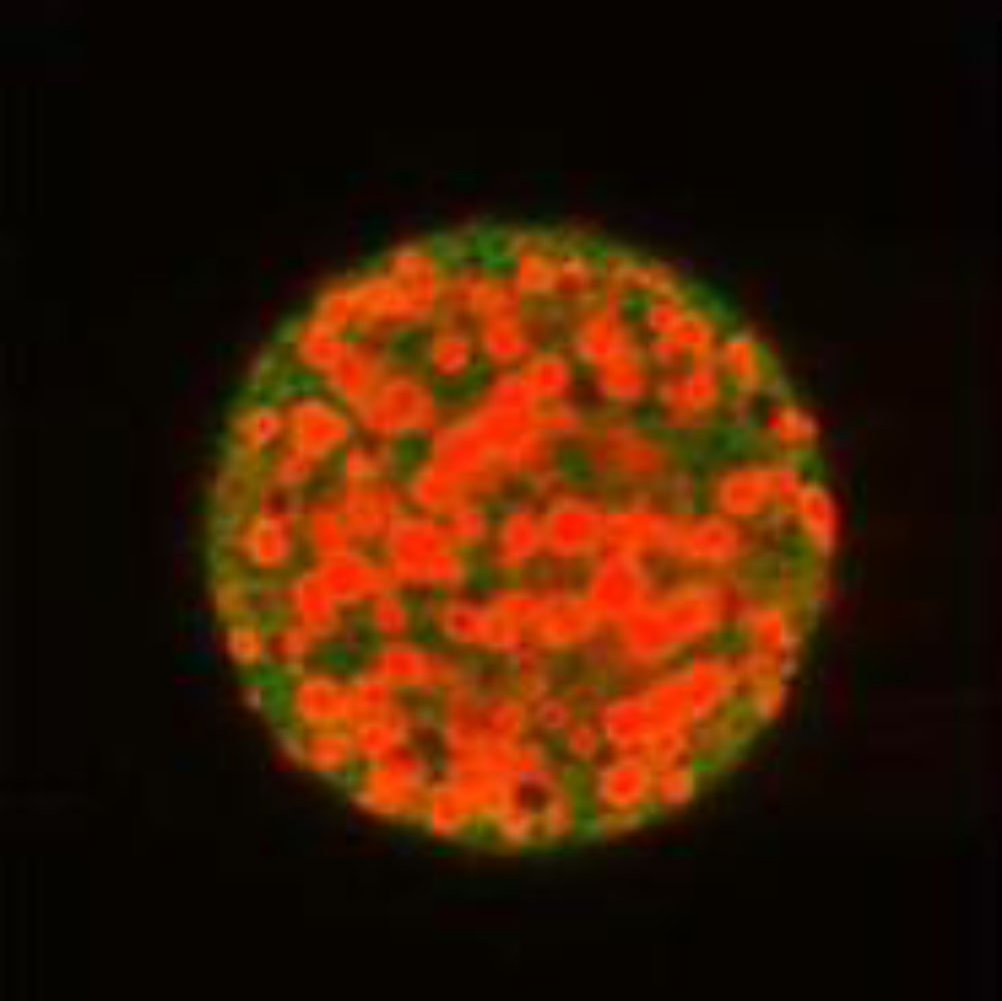
**PLGA particles loaded with therapeutic**. PLGA particles (50:50 lactic to glycolic), labeled with 6-coumarin and loaded with rhodamine labeled PF-4/CTF.

In our lab, PLGA has been used to produce particles loaded with PEX, a fragment of matrix metalloproteinase (MMP)-2, or platelet factor 4 fragments (PF-4/CTF) for the delivery of these angiogenesis inhibitors to glioma bearing nude mice (Figure 2). PEX was detected and isolated from the culture medium of several cell lines and acts as inhibitor of angiogenesis, cell proliferation and migration, demonstrating a 99% suppression of glioma tumor growth in human glioma xenografts [13]. PF-4 is a strong anti-angiogenic factor that inhibits angiogenesis by blocking FGF-2 binding to endothelial cells [16,53]. PEX and PF-4/CTF administered in this mode showed 88% and 95% reduction in tumor volume 30 days post treatment, respectively, demonstrating the advantage of PLGA microspheres for angiogenesis inhibitor delivery to glioma tumors [49].

Other drugs and proteins encapsulated in PLGA microspheres for the treatment of glioma include temozolomide [54], paclitaxel [55], imatinib mesylate for treating intracranial glioma xenografts [56], BCNU as an alternative for Gliadel [57], cisplatin [58], mitoxantrone [59] interleukin-18 [60] and 5-Fluorouracil [61,62].

### Wafers

Wafers are composed of biodegradable polymers that deliver the drugs into brain tumors via local administration, thus, bypassing the BBB. Such device may also be used for the delivery of angiogenesis inhibitors alone or in combination with other chemotherapeutic drugs.

The first device approved by the FDA was Gliadel^®^, a polymeric wafer designed for the delivery of carmustine [63]. Gliadel^® ^is placed directly in the brain cavity created by the resection of the tumor. Clinical studies with Gliadel have shown increased survival rates in newly diagnosed malignant gliomas patient particularly when combined with a chemo-treatment [64,65]. Due to the fact that not all of GBM patients are responsive to carmustine there is a need to evaluate other drugs for the treatment of GBM patients [66].

### Cell delivery platform

Different studies have attempted to use cells, particularly stem cells, that are engineered to secret inhibitors of angiogenesis for brain tumor therapy [67,68]. Another approach, which is based on cell delivery, is polymeric cell encapsulation. The encapsulation system consists of viable cells surrounded by a non-degradable, selectively permeable barrier that physically isolates the transplanted cells from host tissue and the immune system. This platform relies on host homeostatic mechanisms for the control of pH, metabolic waste removal, electrolytes and nutrients. One of the most studied cell micro-encapsulation methods has been based upon alginates, which are polysaccharides extracted from various species of brown algae (seaweed) and purified to a white powder. The alginates have different characteristics of viscosity and reactivity based on the specific algal source and the ions in the solution. Alginate has also hydrophilic properties, which minimize protein adsorption and cell adhesion, thus exhibit a high degree of biocompatibility. For cell encapsulation, the alginate gel is further complexed with polycations such as Poly-L-Lysine (PLL) to form a semi-permeable membrane, which allows the delivery of different bioactive substances to the surrounding while preventing the diffusion of antibodies and other components of the immune system. Cell encapsulation has been used for broad therapeutic applications such as delivery of neuroactive agents for the treatment of age-related degeneration [69,70], Alzheimer's disease[71-74], amyotrophic lateral sclerosis [75,76], neuroprotection [77], Huntington's disease [78,79] and Parkinson's disease[80-82]. This approach has also been used to deliver inhibitors of angiogensis to glioma tumors. Read *et al*. showed that human fetal kidney 293 cells expressing endostatin, an anti-angiogenic 20 kDa fragment of collagen XVIII, encapsulated in sodium alginate, and intracerebral injected near BT4C glioma bearing rats prolonged the survival of the animals by 84% due to induction of apoptosis, hypoxia and large necrotic avascular areas [24,83]. Endostatin released from such delivery system, reduced the functionality and the diameter of blood vessels as well as tumor cell invasion as shown by intravital microscopy [84]. Bjerkvig *et al*. also used the same methodology to deliver endostatin into rat brains [85].

**Figure 3:**
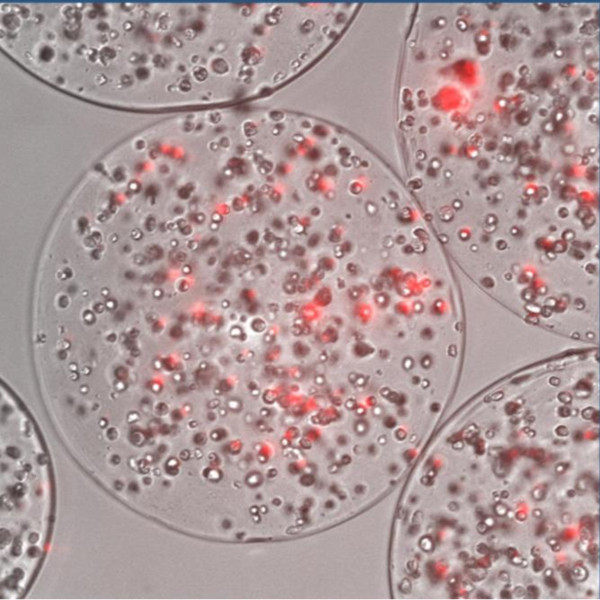
**Cell encapsulation platform**. Human mesenchymal stem cells (hMSCs) labeled with the mCherry fluorescence marker engineered to express PEX and encapsulated in alginate capsules.

Joki *et al*. demonstrated the encapsulation of baby hamster kidney cells (BHK-21) engineered to secrete human endostatin, for the inhibition of glioblastoma xenograft in nude mice [24,86]. In our lab, Goren *et al*. showed that encapsulation of human mesenchymal stem cells (hMSCs), known to be hypo-immunogenic, within alginate-PLL micro-capsules (Figure 3), led to a 3-fold decrease in cytokine expression making them the cell of choice for micro encapsulation cell based-therapy. In this system, the hMSC were genetically modified to express PEX and their injection adjacent to glioblastoma bearing nude mice had led to 87% and 83% reduction in tumor volume and weight, respectively [87]. Feasibility of encapsulated cells to produce single-chain TRAIL has been shown by Kuijlen *et al. *using intracerebral implantation of these capsules in mice brains [88]. Cell encapsulation for CNS malignancies is reviewed in more detailed by Visted *et al *[89].

### Convection-enhanced delivery

Convection-enhanced delivery (CED) was developed to overcome poor brain drug distribution. CED uses hydrostatic pressure to deliver drugs via a catheter located within or around a tumor. As poor drug diffusion through the brain interstitium restricts intratumoral drug administration, fluid convection within the brain (under pressure gradient), can greatly enhance the distribution of molecules in the tumor area. Distribution of drugs via CED is not restricted to white matter and penetration into gray matter can be observed 24 hours after infusion [90]. Nonetheless, Real-time monitoring of CED variability in efficacies as well as limited distribution of the drug still need to be addressed [91].

Saito *et al*. used CED to deliver topotecan entrapped in nano-liposomes (liposomes as drug delivery system will be further discussed in this review in a separate section). CED of liposomal topotecan exerted strong anti-angiogenic activity and disruption of tumor vessels. This delivery platform enabled inhibition of angiogenesis at low concentrations of the therapeutic and demonstrated the ability to deliver anti-angiogenic drugs via CED systems [92]. In another study, Ohlfest *et al*. used CED to co-deliver soluble vascular endothelial growth factor receptor (sFlt-1) and angiostatin-endostatin fusion gene transposons into intracranial glioma model achieving anti-angiogenic effect [93].

## Systemic drug delivery platforms

Systemic drug delivery to the brain requires the consideration of the BBB as previously discussed. More over, systemic delivery systems need to overcome other obstacles such as protein adsorption, enzymatic digestion and engulfment of the delivery particles by phagocytic cells when using 300 nm-10 micrometer particle sizes. Nonetheless, systemic administration can have the benefit of non-invasiveness when compared to local administration using intracranial surgery. Different delivery systems have been developed to achieve systemic therapeutic targeting to the brain including pegylation of drugs, liposomes, polymeric nano-particles, dendrimers and bionanocapsules. We will discuss some of these designed nano-sized delivery systems, which may enable the delivery of drugs to the brain via systemic administration.

### Drug Pegylation

One simplified approach designed to bypass the BBB is the modification of a drug by a polymeric composite, which some term as nano vehicles and other as drug conjugates. One example is the pegylation of interferon-alpha, which is known for its anti-angiogenic effects on tumors and other angiogenic diseases such as AIDS-related Kaposi sarcoma [46]. The motivation behind the pegylation of interferon-alpha was to both reduce its neurologic and immune system toxicities and to improve its circulation time [46,48,94,95]. Pegylation of interferon alpha resulted in a long-lasting form of interferon that may target angiogenesis in glioma [94]. Pegylation has also been used on camptothecin and doxorubicin, improving their solubility, circulation time and lowering their toxicity [96,97].

### Liposomes

**Figure 4:**
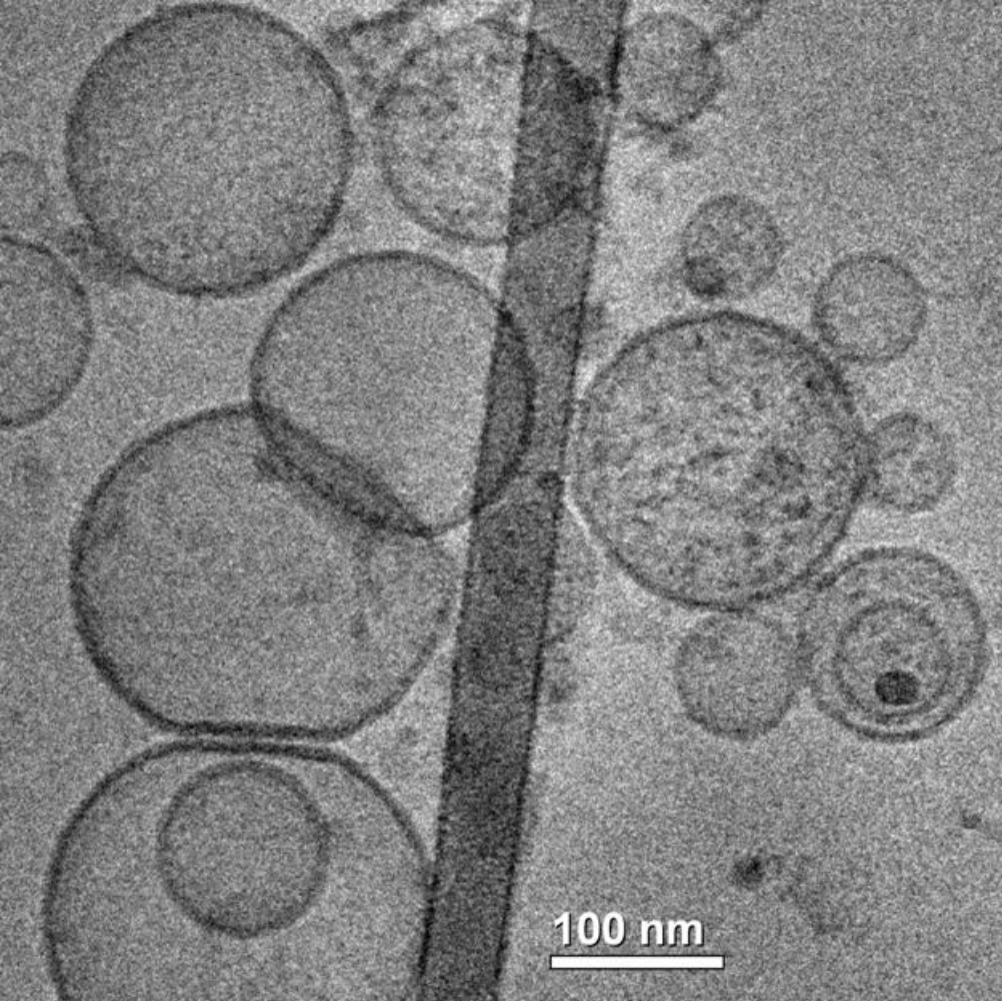
**Liposomes**. TEM image of liposomes made of DOPE -1,2-dioleoyl-sn-glycero-3-phosphoethanolamine, DMPA -1,2-dimyristoyl-sn-glycero-3-phosphate, POPE - 1-palmitoyl-2-oleoyl-sn-glycero-3-phosphoethanolamine and POPC - 1-palmitoyl-2-oleoyl-sn-glycero-3-phosphocholine.

Liposomes are one of the most popular nano-system designs for systemic drug delivery. Liposomes are defined as delivery vehicles composed of phospholipids bilayers (one or more) ranging from tens to hundreds of nanometers in diameter (Figure 4). Liposomes' structure enables the entrapment of water soluble drugs at the aqueous core of the system, while hydrophobic drugs can be entrapped in the lipid bilayer composed of synthetic or natural lipids [98]. Liposomes have been widely researched for their ability to deliver proteins [99], chemotherapeutics [100,101], RNA [102,103], DNA [104] and other therapeutics. Their advantages include biocompatibility, low toxicity, enhanced efficacy of the encapsulated drugs and reduced side effects [105]. Fundamental disadvantage of conventional liposomes is their rapid removal from blood circulation by the mononuclear phagocyte system (MPS). Although this characteristic can be exploited to deliver drugs into phagocytic cells, it hampers the liposomal abilities to target the therapeutic to other cells and organs[106]. Liposomal clearance from the blood circulation is due to recognition of surface bounded opsonins by the MPS [107,108] and membrane lysis of charged liposomes by complement components [109]. One approach to extend liposome circulation time and bypass such fast blood clearance, is to anchor Poly(ethylene glycol) (PEG), to the liposomal membrane- rendering them as stealth liposomes [110]. Adding PEG to the liposome preparations also decreases aggregation and reduces interactions with plasma proteins thus increases their circulation time [111,112]. In gliomas, the BBB is disrupted at the site of the malignant lesion and the leaky endothelium enables passive convective transport of liposomes into the brain. Studies show that stealth liposomes extravasate into the extracellular space forming clusters and acting as a reservoir within the tumor area [113,114]. Caelyx^® ^is a novel formulation of stealth^® ^liposomal doxorubicin. A study with 10 patients with metastatic brain tumors and five patients with brain glioblastoma undergoing radiotherapy confirmed intense accumulation of radio-labeled Caelyx^® ^in the brain tumor as compared to the normal brain tissue[115]. In another study, doxorubicin encapsulated in liposomes exhibited break down of tumor vasculature [116]. Other drugs encapsulated in liposomes for treating brain tumors include taxol [117] and arsenic trioxide which down-regulated the expression of VEGF [118].

### Polymeric nano-particles

Polymeric nano-particles (1-999 nano-meters) have attracted much attention as vehicles for systemic drug delivery due to their biocompatibility and stability properties. The diversity of materials, mostly synthetic, used to formulate nano-particles include poly(butyl cyanoacrylate), Poly(ethylene-glycol), Poly(lactic-co-glycolic) acid, Poly-glycerol and others [45-48]. Hekmatara *et al*. showed that doxorubicin bound to polysorbate-coated nano-particles made of Poly(butyl cyanoacrylate) and injected intravenously into tumor bearing Wistar rats, had a drastic effect on vessel density with neither neuronor systemic toxicity [45]. Another approach that might be feasible for brain tumor targeting is a novel delivery system termed 'nanocell'. This delivery system is composed of nuclear nano-particle enveloped with pegylated-phospholipid block-copolymer [95]. The idea behind this system is to deliver inhibitors of angiogenesis followed by the delivery of cytotoxic drugs into the tumors. In this system, the angiogenic inhibitor is located within the lipid layer and the chemotherapeutic drug is incorporated to the nano-particle. Sengupta *et al*. showed that doxorubicin conjugated to the copolymer poly-(lactic-co-glycolic) acid (PLGA) enveloped within PEG distearoylphosphatidyl-ethanolamine (PEG-DSPE), phosphatidylcholine and cholesterol in an optimal ratio with combretastatin (which causes rapid vascular shutdown) had significant effect on tumor vasculature [95]. Another interesting approach for targeting angiogenesis is the use of RNA-based inhibitors [31]. Glioma angiogenesis has being targeted by RNA-based inhibitors, which include short hairpin RNAs (shRNAs) and short interfering RNAs (siRNAs) against urokinase-type plasminogen activator and MMP-2 respectively [119-121]. These studies showed significant anti-angiogenic effect and impaired glioma invasion in mouse models. Recently, microRNAs (miRNAs), a new group of RNA inhibitors, have attracted much attention as unlike siRNAs and shRNAs they can interact with many mRNAs due to incomplete nucleotide complementarities [122]. One such group of miRNAs was related to angiogenesis [123]. Ofek *et al*. developed a novel polymerized polyglycerol-based cationic dendrimer core shell structures, which can deliver siRNAs to cells and inhibit angiogenesis [47]. These siRNA-dendrimers improved the stability, uptake and intracellular trafficking of siRNAs demonstrating *in-vivo *targeting of luciferase in luciferase-expressing tumors in mice [47]. In another study, Schneider *et al*. showed that poly-(butyl cyanoacrylate) nano-particles coated with Polysorbate-80 were capable of delivering antisense oligonucleotide, specifically against TGF-β2, into intracerebral tumors via the BBB [48,124].

A different concept of nano-delivery system has been recently introduced and termed 'bionanocapsules' [125]. Bionanocapsulses are hollow nano-particles with an average diameter of 80 nm, displaying specific affinity to human hepatocytes via the pre-S1 peptide displayed on their surface and have successfully delivered peptides, genes and siRNA to the liver [125]. Tsutsui *et al*. demonstrated that the deletion of the pre-S1 peptide and conjugation of the anti-human EGFR, recognizing EGFRvIII, abolished hepatocytes targeting, making them capable of targeting brain tumor *in-vitro *and *in-vivo *[126]. These studies suggest a new drug delivery system for brain tumor and possible use of such delivery systems for the delivery of anti-angiogenic drugs should be considered.

## Optimizing therapeutic delivery using bio-mathematical models

Mathematical models are based on the optimization of natural algorithms within the growing biomathematical tool kit and can provide a deeper understanding of the dynamic biological process involved with tumor angiogenesis, tumor growth or other tumor properties. Using mathematical modeling, tumor behavior upon drug treatment (free or released), from a delivery platform can be predicted. This is a powerful tool that can minimize invasive protocols, reduce drug quantities, force combinational drug treatments and reduce the number of animals required for *in-vivo *experiments.

These methodologies have already been applied in the fields of cancer chemotherapy and cancer immunotherapy with minimization of tumor burden as their primary objective [127-130] and may be of great importance when considering GBM treatments. Gevertz *et al*. described for the first time, a biomathemathical model for the follow up of GBM growth, which is based on the evolution of the tumor microvasculature and mass. In this model they assume that the key players in glioma angiogenesis are VEGF, Ang-1 and Ang-2 (Angiopoietins) and were able to show that an "angiogenic switch" is valid, just as Folkman predicted [131,132]. Kronik *et al*. successfully created a mathematical model which describes the growth rate of high grade gliomas post stimulation of alloreactive CTLs. The mathematical analysis shows that differences in the growth rate parameter alone suffice for explaining the difference in the success of cellular immunotherapy treatment for grade III and grade VI glioma patients [133].

In our lab, Benny *et al*. presented a pre-clinical study that used PLGA microspheres loaded with imatinib mesylate in GBM model in mice [56]. Together with Kronic *et al*., we were able to extract, using mathematical modeling, the pharmaco kinetics of imatinib mesylate, which correlated significantly with the kinetics obtained experimentally (unpublished data). The models allow us to predict the release of drug in the intracranial environment, its clearance and the therapeutic window needed for additional administration of the loaded PLGA particles.

## Conclusions

Targeting brain tumor angiogenesis is a promising approach to arrest tumor growth. Nonetheless, clinical studies with inhibitors of angiogenesis have produced disappointing results. This may be due to the fact that these inhibitors were administered alone, without standard chemo and radiotherapy. It is also strongly possible that the way of administration (mostly intravenously), drug stability and quantities needed to achieve therapeutic outcome, hamper the therapeutic potential of such group of drugs. More over as the brain is a unique organ, protected via the BBB and efflux transporters (although disrupted in high grade glioma patients), the drugs need to bypass such barriers and reach the tumor in a therapeutic dosage. Therefore developing drug delivery platforms that bypass such barriers and target the therapeutics to the tumor site and on the other hand stabilize and protect the drug until it is released near or in the tumor bed can result in surprising therapeutic effects for such group of inhibitors.

The progress made in the field of biomaterials together with the pharmaceutical field, contribute vastly to the development of such local and systemic delivery platforms.

Yet, regardless of the enormous advantageous that these technologies can offer, the road to clinical studies using these platforms is still facing problems, which need to be studied and solved as they target the most protected and closed organ, the brain. Selection of polymers, preparation methods, drug loading and release profile, clearance of the drugs and immune aspects need to be taken in account and studied carefully to pave the way for new and promising delivery platform to reach the clinical setting.

## Competing interests

The authors declare that they have no competing interests.

## Authors' contributions

GA and MM have both contributed to the preparation of this manuscript. All authors read and approved the final manuscript.
